# The Entrapment Ability of Aqueous and Ethanolic Extract of *Teucrium Polium*: Glucose Diffusion into the External Solution

**Published:** 2013

**Authors:** Durdi Qujeq, Ali Babazadeh

**Affiliations:** 1 *Cellular and Molecular Biology Research Center (CMBRC), Babol University of Medical Sciences, Babol, Iran.*; 2 *Faculty of Medicine, Babol University of Medical Sciences, Babol, Iran.*; 3 *Department of Biochemistry and Biophysics, Faculty of Medicine, Babol University of Medical Sciences, Babol, Iran.*

**Keywords:** Dialysis tube, diffusion, glucose, extract, *Teucrium polium*

## Abstract

Some plant extracts showed the ability to retard the diffusion of glucose across the dialysis tube. The present study was designed to investigate the effect of aqueous and ethanolic extracts of *Teucrium polium* (*T. polium*) on glucose movement across the dialysis tube. The *T. polium* powder was dissolved in ethanol and distilled water. Then glucose was added to make a final concentration of 0.2 – 0.8 g/l glucose with aqueous or ethanolic extract of *Teucrium Polium*. Fifteen milliliter of each concentration (0.2 – 0.8 g/l) of glucose was dialyzed against 50 ml of distilled water at 20 ^◦^C in a dialysis tubing cellulose membrane (molecular weight cut off = 10000 Da) every 4 h for 24 hours under rotationally shaking. The released glucose was determined by glucose oxidase kit. Aqueous extract of *T. polium* did not show any significant effect on the glucose movement. But, ethanolic extract of *T. polium* was found to exhibit a significant stimulation on glucose movement from dialysis tube to the external medium. Our findings suggest the possible importance of other factors besides viscosity in determining the anti- diabetic behavior of *T. polium*.

Previous researches have indicated that in experimental animal models, the aqueous extract of *T. polium* exhibited antidiabetic and hypolipidemic effects (1, 2). Most of these effects have been related to the chemical components of *T. polium* (3, 4). In recent years, investigators have studied the influence of different plant extracts on the diffusion of glucose across the semi-permeable membrane or dialysis tube (5, 6). Dialysis tubing technique is a simple model to evaluate the potential of soluble dietary fibers to additionally retard the diffusion and movement of glucose in the intestinal tract (7). It seems that the movement in this system is not by true diffusion but is assisted by the convective activity of intestinal contractions (8). There are some experimental evidences suggesting that the retardation of the nutrient flow into the external medium is an indication of the modulating effect of that fiber on glucose absorption in the intestine (7, 8). Many investigators identified by gas chromatographic and spectroscopic techniques, beta sitosterol, stigmasterol, campesterol, brassicasterol and clerosterol in the *T. polium* (9). Previous studies also established that *T. polium* has traditionally been used as herbal anti-diabetic medicines (10).

Pancreas regeneration is also favorized by *T. polium* (11). Some data have revealed that traditional medicine can be hepatotoxic (12). The *T. polium* extract modulates the serum, liver and muscle triglyceride content and improves the insulin resistance in the experimental animal (13). The purpose of this study is to investigate the mode of action of aqueous and ethanolic extracts of *T. polium* on glucose diffusion across the dialysis tube.

## Materials and Methods

Glucose kit was from Pars Azmoon Co., Tehran, Iran. Dialysis tubing cellulose membrane (molecular weight cut off = 10000 Da.) was from Medical Industries, Tehran, Iran. All other chemicals were analytical grade.

**Plant material**

The flowering aerial parts of *T. polium* were collected from a local store. The plant was authenticated by the center for agricultural research and natural resources of Mazandaran, Faculty of Agriculture of University of Mazandaran. The plant material was rinsed from dust by tap water and dried under shade at room temperature for four days. The dried plant material was grounded into fine powder using an electric grinder and was used as such in the subsequent experiments.

**Preparation of** ***T. polium*** **extract**

Fifty grams of the powdered aerial part of the plant was placed in one litter of distilled water or ethanol. The mixture was shacked for 1 h, then boiled for 1 h. The extract was filtered through a mesh and a whatman paper No. 2. The yield of this operation was 1/8 of the starting plant powder. The extraction steps were repeated several times to obtain enough material to be used in all experiments. The powder was kept in a refrigerator (– 20 ºC) until use.

Plant powder samples were dissolved in water, precipitated twice with 2 volumes of 70% ethanol and dialyzed against distilled water in a dialysis tubing cellulose membrane (molecular weight cut off = 10000 Da). 

**Effect of Aqueous extracts of** ***T. polium*** **on in vitro glucose movement**

In vitro glucose movement investigation was carried out according to the method described by Edward et al. (8) with some modification. Briefly, this system consisted of a dialysis tubing 15 cm long piece of 1 cm large (MW 10000), that has been soaked in distilled water. Then one end of the tubing was tied off to form a bag. To open the other end of the bag, we rubbed the end between fingers until the edges separate. Then 0.20 – 0.80 g glucose (Sigma,USA) and plant extract was placed into a one - sided sealed dialysis tube, after which the other end was sealed and the tube was placed in a glass beaker containing 50 ml distilled water, leaving sufficient space for the expansion of bags content. Then the beaker was placed in a shaker (Kottermann model 3165, Germany) at 20 ºC. The diffusion of the glucose into the external medium was monitored at set time intervals 0, 4, 8, 12, 16, 20 and 24 hrs with respect to a negative control (water).

**Glucose concentration assay**

All tests were carried out in triplicate and glucose content was measured spectrophotometri--cally (Jenway, Model 6505, UK) using the glucose oxidase kit (Pars AzmmonCo, Tehran, IRAN).

**Statistical analysis**

All values have been presented as mean ± SD. Statistical analysis was done using SPSS version 18. The significance of differences between the mean values was determined by analysis of variance (ANOVA), and a p-value of less than 0.001 was considered statistically significant.

## Results

**Figure 1:**
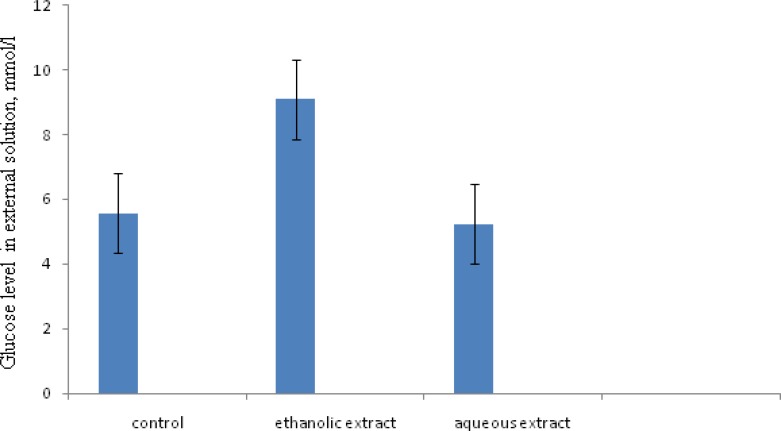
Effect of different extracts of *T. polium* ( 0.8 g/l at 20h ) on the movement of glucose out of the dialysis tube, glucose level in external solution. Values were expressed as Mean ± SD of three determinations. (Column 1; control. Column 2; alcoholic extract . Column 3; aqueous extract , P<0.001

In this study, a solution of glucose was placed inside a bag of dialysis tube and transferred into a baker containing distilled water. Our results revealed that, aqueous extract of *T. polium* did not show any significant effect on glucose movement after 0, 4, 8, 12, 16, 20 and 24 hrs. But, under the influence of ethanolic extracts of *T. polium*, glucose diffusion was significantly increased. Figure 1 shows that the retardation effect on glucose movement of the ethanolic extract of *T. polium* ( 0.8 g/l at 20h ) was lower compared to aqueous extract of *T. polium*.

## Discussion

This study was undertaken to investigate the contribution of *T. polium* extracts with respect to its glucose retardation activity across the dialysis tube by taking pure water as negative . This study highlighted the mode of action of aqueous and ethanolic extracts of *T. polium* regarding glucose diffusion in an in vitro model. In this regard, dialysis tubing technique is a simple model to evaluate the potential of plant extracts to additionally retard the diffusion of glucose in the intestinal tract. Movement in this system is assisted by the convective activity of intestinal contractions (5, 8).

The present work shows that the effect of aqueous extracts of *T. polium* was very close to the control. It did not show any significant effect on glucose movement (Fig. 1). It was evident from figure 1 that aqueous extract was not a potent inhibitor of glucose diffusion.

Ethanolic extracts of *T. polium* increased the movement of glucose. The highest retardation effect of *T. polium* appeared at time point of 20 h and the concentation of 0.8 g/l of glucose. Our results indicated that at the beginning of dialysis, diffusion of glucose was slow. Our results show that *T. polium* did not show any entrapment ability in decreasing glucose movement into the external solution. Thus, the anti-diabetic action of *T. polium* is not likely to be related to glucose diffusion. Anti-diabetic activity of aqueous and ethanolic extracts of *T. polium* may depend on other mechanisms. The stimulation of glucose transport observed in the present study is explained by an increase in diffusion from the dialysis tube. The results in this study were in agreement with previous works showing that plant extracts had different role on glucose diffusion (5, 6). Further studies need to be conducted in order to confirm the in vivo action of *T. polium* with respect to the movement of glucose.

While the small sample size of the study limit the generalizability of the results, future research with an expanded sample size, can yield important new insights into preventive dietary strategies. Despite the limitations of this *in vitro *study, there seemed to be various mechanisms possibly involved by aqueous and ethanolic extracts of *T. polium* due to their anti-diabetic properties. Further studies are also needed to check the role of viscosity of aqueous and ethanolic extracts of *T. polium* on glucose movement.
